# Antibiotic resistance and frequency of class 1 integrons among *Pseudomonas aeruginosa*, isolated from burn patients in Guilan, Iran

**Published:** 2013-03

**Authors:** Iraj Nikokar, Azita Tishayar, Zinab Flakiyan, Kobra Alijani, Saeedeh Rehana-Banisaeed, Mojtaba Hossinpour, Sirous Amir-Alvaei, Afshin Araghian

**Affiliations:** 1 Laboratory of Microbiology and Immunology of Infectious Diseases, Paramedicine Faculty, Guilan University of Medical Sciences,Langeroud, Iran; 2 Rasht Burn Center, Guilan University of Medical Sciences, Rasht, Iran; 3 Reference Laboratory, Guilan University of Medical Sciences, Rasht, Iran

**Keywords:** Antibiotic resistance, *Pseudomonas aeruginosa*, Integron, Burn Wound Infection

## Abstract

**Background and Objectives:**

Antibiotic resistance of *Pseudomonas aeruginosa* remains a major problem in burn patients. The main objective of this study was to determine the antibiotic resistance pattern and frequency of class 1 integrons among *P. aeruginosa* strains isolated from patients with burn wound infections in a new Burn Centre in Guilan, Iran.

**Materials and Methods:**

The bacterial isolates were collected from 182 patients with burn wound infections and *P. aeruginosa* species were identified by standard bacteriological methods. The drug susceptibility test, using 11 antimicrobial agents, was performed for all the isolates via agar disk diffusion method. PCR was carried out for the detection of integrons.

**Results:**

Out of a total of 182 hospitalized patients in the burn center assessed, 86 (47%) found to have *P. aeruginosa* in their isolates. Resistance rates to various antibiotics were as follows: cloxacillin (91.8%), cotrimoxazole (86%), cephazolin (83.7%), carbenicillin (74.4%), piperacillin (69.9%), ceftazidime (68.8%), ciprofloxacin (66.3%), tobramycin (58.2%), amikacin (48.8%) and gentamicin (37.2%), while the most effective antibiotic was imipenem with a resistance rate of 23.3%. Thirty nine (45.3%) isolates were detected as multi-drug resistant. The PCR results showed that 37 (43%) *P. aeruginosa* isolates and 27 (69.2%) multi-drug resistant strains harbored class 1 integrons. A significant correlation was obtained between the presence of integrons and resistance against imipenem, ceftazidime, piperacillin and ciprofloxacin (P <0.001).

**Conclusion:**

Optimization of using antimicrobial agents and control of infection is recommended to prevent the increasing population of drug resistant organisms in the new burn centre setting in this study. Furthermore, the high frequency of class 1 integrons among multi-drug resistant strains might be responsible for dissemination of antibiotic resistance gene.

## INTRODUCTION

*Pseudomonas aeruginosa*, as one of the most common microorganisms worldwide, is a leading cause of nosocomial infections, especially among burn patients (1). Extensive burns can contribute to the suppression of immune system and provide a suitable site for multiplication of *P. aeruginosa* (2). This organism contaminates floors, bed rails, sinks of burn hospitals, and is frequently isolated as an opportunistic pathogen from recurrent infections of hospitalized patients (3). Microorganisms may also be transferred to a patient's skin surface via contact with contaminated external environmental surfaces and the hands of health care workers (1). Antibiotic resistance is a worldwide emerging problem, and the widespread use of antibiotics is likely the main reason for the increase in the drug resistance among *P. aeruginosa* strains (4). In the recent years, *P. aeruginosa* has developed severe infections in patients with burn injuries and demonstrated increased resistance to various antimicrobial agents (3, 5). Besides, in many bacteria, exchangeable genetic elements such as plasmids, transposons and integons are responsible for the dissemination of antibiotic resistance (6). Integron structure contains essential elements for insertion and mobilization of gene cassettes. Resistance gene cassettes in class 1 integrons are associated with multi-drug resistance among Gram negative bacteria as well as *P. aeruginosa* (7). Previous studies have shown that *P. aeruginosa*, with multi-drug resistance patterns, are widespread among Iranian hospitals (8, 9). According to the literature review, the current research is the first study to assess the antibiotic resistance patterns and frequency of integrons among *P. aeruginosa* strains isolated from burn wound infections. The results of this study can be quite important for choosing an efficient approach to managing infection in the burn hospital of the Guilan province.

## MATERIALS AND METHODS

In a cross–sectional study, microbiological wound swabs were collected from 182 patients with clinical signs and symptoms of burn wound infection in a teaching burn hospital in Guilan province, between February 2010 and January 2011. These swab specimens, were first inoculated onto Tryptic Soy Broth (TSB) and the primary isolation was performed on blood agar, McConkey agar and eosin methylene blue agar media. *P. aeruginosa*, isolated from wound injuries, was identified by standard bacteriological methods including: Colony morphology, Gram staining, pyocyanin pigment production, growth at 44°C, Catalase and oxidative-fermentative (OF) tests. The drug susceptibility test was carried out for all the isolates on Mueller-Hinton plates and zones of inhibition were measured by Kirby-Bauer agar disk diffusion method in accordance with the recommendations of clinical and laboratory standards institute (CLSI). The antimicrobial agents used in this test were as follows: amikacin (30 ug), ceftazidime (30 ug), cephazolin (30 ug), cloxacillin (5 ug), carbenicillin (30 ug), gentamicin (10 ug), imipenem (10 ug), piperacillin (100 ug), ciprofloxacin (5 ug), tobramycin (10 ug), and cotrimoxazole (25 ug) (Mast Diagnostics, Mast Group Ltd, Merseyside, UK). *Pseudomonas aeruginosa* ATCC 27853 was used as the control organism. The results were interpreted as susceptible, intermediately susceptible or resistant by measuring the diameter of inhibition zone, according to the criteria designated by CLSI (10). In this study, multi-drug resistant isolates were defined as those resistant against at least three of the four following groups: (1) imipenem or meropenem; (2) cefepime or ceftazidime; (3) piperacillin, piperacillin–tazobactam or ticarcillin–clavulanic acid and (4) ciprofloxacin or levofloxacin (9, 11). The isolates were non repetitive meaning that each patient was sampled only once. All the isolates were screened by PCR for class 1 integrons using the primers 5'CS (GGCATCCAAGCAGCAAG) and 3'CS (AAGCAGAC TT GA CCTGA). In the this study analysis of the integrase gene was carried out for the detection of class 1 integrons, as reported by Levesque et al (12). Amplification was performed for 35 cycles as: initial denaturation at 94°C for 5min, denaturation at 94°C for 1min, primer annealing at 57°C for 1min, extension at 72°C for 2min and final extension at 72°C for 5min. The PCR products were analyzed by acryl amid gel electrophoresis. Analysis of the findings was performed using Chi Square test and Statistical Package for Social Sciences (SPSS, version 16). The study was confirmed by the ethical committee of the Guilan University of Medical Sciences.

## RESULTS

**Fig. 1:**
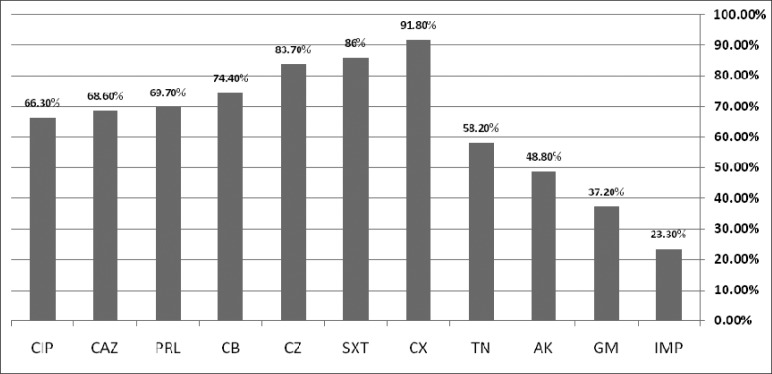
The percentage rate of antibiotic resistance and presence of class 1 integrons among 39 MDR *P. aeruginosa* strains isolated from patients hospitalized in a burn center in Guilan, Iran. Amikacin (AK), ceftazidime (CAZ), cephazolin (CZ), cloxacillin (CX), Carbenicillin (CB), Gentamicin (GM), Imipenem (IPM), Piperacillin (PRL), Ciprofloxacin (CIP), Tobramycin (TN) and Cotrimoxazole (SXT)

Out of 182 hospitalized patients in the burn center assessed, 86 (47%) found to have *P. aeruginosa* in their isolates. Table 1 shows the antibiotic susceptibility pattern of the *P. aeruginosa* isolates indicating that the isolates have acquired high level resistance against cloxacillin (91.8%), cotrimoxazole (86%), cefazolin (83.7%), carbenicillin (74.4%), piperacillin (69.7%) ceftazidime (68.6%), and ciprofloxacin (63.3%). A low level resistance was recognized for imipenem (23.3%) and gentamicin (37.2%), while an intermediate level resistance was found against the amikacin (48.8%) and tobramycin (58.2%) (Fig. 1). A total of 17 (19.7%) isolates were resistant against all the tested antibiotics, 39 (42.3%) showed resistance against at least three of the four following antibiotics: (1) imipenem, (2) ceftazidime (3), piperacillin, and (4) ciprofloxacin. These isolates were defined as multi-drug-resistance (MDR) (Table 2). The PCR results revealed that 37 (43%) *P. aeruginosa* isolates harboured class 1 of integrons. Among the multi-drug resistance (MDR) strains, 27 (69.2%) showed class 1 integrons and a significant correlation was found between the presence of integrons and resistance aganist imipenem ceftazidime, piperacillin, and ciprofloxacin (P < 0.001).

**Table 1:** Antibiotic susceptibility patterns of *P. aeruginosa* isolated from patients hospitalized in a burn center in Guilan, Iran.

Antibiotics	Sensitive		Intermediate		Resistance	

	n	%	n	%	n	%
**Imipenem**	64	74.4	2	2.3	20	23.3
**Gentamicin**	52	60.5	2	2.3	32	37.2
**Amikacin**	41	47.7	3	3.5	42	48.8
**Tobramycin**	36	41.8	0	0	50	58.2
**Ciprofloxacillin**	28	32.5	1	1.2	57	63.3
**Ceftazidime**	23	26.7	4	4.7	59	68.6
**Piperacillin**	24	27.9	2	2.3	60	69.7
**Carbenicillin**	22	25.6	0	0	64	74.4
**Cephazolin**	14	16.3	0	0	72	83.7
**Cotrimoxazole**	11	12.8	1	1.2	74	86
**Cloxacillin**	7	8.2	0	0	79	91.8

**Table 2:** Characteristics of multi-drug resistance (MDR) isolates and the presence of class 1 integrons among *P. aeruginosa* strains isolated from patients hospitalized in a burn center in Guilan Iran.

Characteristics	No. of isolates (%)	Integron positive	Integron negative	P. value
A) *MDR Isolates	39 (45.3%)	27(69.2%)	12 (30.8%)	< 0.001
**B) Resistance to Antibiotic**				
**Imipenem**	24 (64.1%)	16(66%)	8(33%)	< 0.001
**Ceftazidime**	37(94.8%)	25(67.5%)	12(32.4%)	< 0.001
**Piperacillin**	39 (45.3%)	27(69.2%)	12 (30.8%)	< 0.001
**Ciprofloxacin**	38(97.4%)	27(71.1%)	11(28.95%)	< 0.001

* MDR isolates are resistant to at least three of the following four antibiotics: imipenem, ceftazidime, Piperacillin and ciprofloxacin (9, 11).

## DISCUSSION

*P. aeruginosa* still remains an important opportunistic cause of nosocomial infection and has developed resistance to various antimicrobial agents in burn centers (1, 5). This study was carried out for the first time at a single referral burn center in the Guilan province. The results of this study showed that 86 (47%) isolates contained *P. aeruginosa* that is in agreement with the results of previous studies (13, 14). The results of some studies demonstrated a higher frequency of *P. aeruginosa* among the patients with burn infections especially in many developing countries such as India 59% (15), Turkey 57% (16) and Pakistan 54.4% (17). In Iran, the highest frequency rate was reported as 73%and 70.5% in two old referral burn centers in Tehran, Iran (18, 19). In another study, however, a lower frequency rate of *P. aeruginosa* was reported from a burn hospital in Ahvaz, Iran (20). Resistance to antimicrobial agents is the main problem among *P. aeruginosa* strains isolated from wound infections in burn centers. Many studies have shown that *P. aeruginosa*, particularly the multi-drug resistant strains, are widespread among Iranian hospitals (3, 8). In the current study, high level resistance, among *P. aeruginosa* strains isolated from burn wounds, was detected against the following antibiotics: cloxacillin, cotrimoxazole, cephazolin, carbenicillin, piperacillin, ceftazidime. Previous studies reported resistance against many antibiotics used for treating patient with burn wounds infected by *P. aeruginosa* (21–23). The resistance rate of *P. aeruginosa* isolates against aminoglycoside groups; tobramycin, amikacin, and gentamicin were 58.2%, 48.8%, and 37.2%, respectively. Higher rates of resistance to these aminoglycosides antibiotics, including tobramycin (82.%), amikacin (73%), and gentamicin (80%), was reported by BojaryNasrabadi and Hajia (19). In the present study, imipenem was the most effective antibiotic against *P. aeruginosa* that is in consistent with other studies conducted at Iranian burn centers (24, 25). However, recent studies have shown increasing imipenem resistant strains in Iranian burn care centers (26, 27).

The findings of this study showed that 17 (19.7%) *P. aeruginosa* isolates were resistant to all the classes of antibiotics tested. According to a survey conducted by Kohanteb et al (22), it was found that 26.7% of the *P. aeruginosa* isolates, obtained from patients with burn wound infections, were resistant to all the anti-pseudomonal antibiotics tested.

Different definitions have been employed to characterize multi drug resistant (MDR) isolates of *P. aeruginosa* in biomedical publications (11). In the majority of studies, multi-drug resistance was defined as resistance to at least three drugs out of a variety of antibiotic classes, mainly aminoglycosides, antipseudomonal penicillins, cephalosporins, carbapenems and fluoroquinolones (9). According to a definition offered by Ohmagari N *et al*., (28), 39 (42.3%) of the isolates showed resistance to at least three of the four tested antibiotics including; (1) imipenem (2) ceftazidime, (3) piperacillin and (4) ciprofloxacin. Over the recent years, several reports confirmed an increasing multi-drug resistance among *P. aeruginosa* strain isolated from burn wound infections (3, 19, 21). A comparison made against this study, carried out for the first time in a referral burn centre in Guilan, showed that both the frequency and antibiotic resistance rates were lower than those in two old referral burn centers in Tehran. The high frequency rate of *P. aeruginosa* in the two referral burn centers might be due to the prolonged hospital stay and intensive use of antibiotics. Colonization of resistant *P. aeruginosa* strains, originated from exogenous sources such as contaminated equipments or other patients, can facilitate transmission of resistant bacteria between environment and patients (29). In the present study, Out of the 86 *P. aeruginosa* strains isolated from burn wound samples, 37 (43%) had class 1 integrons, as detected by PCR.

Literature review reveals that very little has been published on the frequency of class 1 integrons among *P. aeruginosa* strains isolated from burn injuries and that the majority of studies are conducted regarding the isolation from different clinical samples. In a study carried out by Yousefi et al (30) on samples such as bronchial fluid, blood culture, catheter, cerebrospinal fluid, ear, pleural fluid, sputum, urine, and wound, 90 (56.3%) of the *P. aeruginosa* isolates carried class 1 integrons. In other studies carried out on different clinical specimens in Amazon area of Brazil and Zhenjiang area of China, 41.5% and 38.0% of *P. aeruginosa* isolates were found to be positive for class 1 integrons, respectively (31, 32).

In the present study, a clear difference was observed in the frequency of class 1 integrons between multi-drug resistance and non MDR strains. Several studies have reported higher prevalence rate of class 1 integrons among the MDR strains of *P. aeruginosa* (30, 33, 34).

Furthermore, a significant correlation was observed between the presence of integrons in the MDR isolates and the antibiotic resistance to imipenems, ceftazidime, piperacillin and ciprofloxacin. These data are in agreement with the findings of Gu et al (33) in china and Yousefi et al. (30) in Iran.

In conclusion, although the frequency and antibiotic resistance rates of *P. aeruginosa* strains were lower, in the present study, compared to other studies conducted in the two referral burn hospitals in Tehran. Optimization of antimicrobial use and control of infection is recommended to prevent the increase in the population of drug resistant organisms in the referral burn hospital in Guilan. Moreover, a significant correlation between the presence of integrons and antibiotic resistance among the MDR isolates suggested that integrons might be responsible for the distribution of antibiotic resistance genes among multi-drug-resistant strains.
